# Extrinsic Calibration of Camera and 2D Laser Sensors without Overlap

**DOI:** 10.3390/s17102346

**Published:** 2017-10-14

**Authors:** Khalil M. Ahmad Yousef, Bassam J. Mohd, Khalid Al-Widyan, Thaier Hayajneh

**Affiliations:** 1 Department of Computer Engineering, The Hashemite University, Zarqa 13115, Jordan; bassam@hu.edu.jo; 2 Department of Mechatronics Engineering, The Hashemite University, Zarqa 13115, Jordan; alwidyan@hu.edu.jo; 3 Department of Computer and Information Sciences, Fordham University, New York, NY 10023, USA; thayajneh@fordham.edu

**Keywords:** calibration, range sensing, mobile robot, mapping, 2D lidar sensor

## Abstract

Extrinsic calibration of a camera and a 2D laser range finder (lidar) sensors is crucial in sensor data fusion applications; for example SLAM algorithms used in mobile robot platforms. The fundamental challenge of extrinsic calibration is when the camera-lidar sensors do not overlap or share the same field of view. In this paper we propose a novel and flexible approach for the extrinsic calibration of a camera-lidar system without overlap, which can be used for robotic platform self-calibration. The approach is based on the robot–world hand–eye calibration (RWHE) problem; proven to have efficient and accurate solutions. First, the system was mapped to the RWHE calibration problem modeled as the linear relationship AX=ZB, where X and Z are unknown calibration matrices. Then, we computed the transformation matrix B, which was the main challenge in the above mapping. The computation is based on reasonable assumptions about geometric structure in the calibration environment. The reliability and accuracy of the proposed approach is compared to a state-of-the-art method in extrinsic 2D lidar to camera calibration. Experimental results from real datasets indicate that the proposed approach provides better results with an L2 norm translational and rotational deviations of 314 mm and $0.12^{\circ }$ respectively.

## 1. Introduction

Accurate extrinsic calibration between different sensors is an important task in many automation applications. In order to improve environmental perception in such applications, multiple sensors are used for sensor data fusion to produce unified and accurate results [1]. Some of those applications can be seen in surveillance and motion tracking, mobile robotics, etc. For example, in mobile robotics, usually a given robotic platform is equipped with single or multiple laser range finders, single or multiple cameras sensors, single or multiple RGB-D sensors, and others.

It is important to compute the relative transformations (i.e., extrinsic parameters) between such different classes of sensors quickly and easily using a standard methodology. This would be more efficient compared with using specialized routines or ad hoc implementations to adapt to a particular application or platform [2,3,4]. Often, a sensor is added to a robotic platform, for the purpose of extending functionality, or existing sensors being disassembled and assembled again after maintenance or replacement. In this case, one should be able to efficiently calibrate the robotic platform without the need of an expert knowledge. In fact, enabling workers in a mall, a workshop, or at an assembly line to calibrate a robotic platform themselves will substantially reduce their workload and calibration time.

Therefore, one of the main motivations of this paper is to present a novel and flexible approach for the calibration of 2D laser range finder (or lidar) and camera sensors of different configurations (e.g., overlapping and non-overlapping field of views of the sensors). Additionally, we aim on exploring and solving the extrinsic calibration problem at hand into possibly other problem domains proven to have efficient and accurate solutions, and that also best suitable for calibrating non-overlapping sensors. To do so, the proposed work interprets the multi-sensor extrinsic calibration problem of a camera and 2D lidar sensors as the well-known robot–world hand–eye (RWHE) calibration problem. The only requirement is the availability of an additional reference frame, which is stationary with respect to the calibration object frame and is detectable in the 2D lidar frame.

The RWHE calibration problem was formulated by Zhuang et al. [5] as the homogeneous matrix equation AX=ZB for calibrating a visual sensor rigidly attached to the end effector of a standard robot arm (The RWHE problem and its mathematical formulation AX=ZB are described and discussed in Section 3). The advantages of the RWHE formulation compared to other state-of-the-art formulations (e.g., [6,7,8]) are threefold. First, the RWHE solution is well investigated in the literature and there are many efficient and accurate algorithms developed for it. Second, it is efficient and accurate to apply the RWHE formulation on the extrinsic 2D lidar–camera calibration, especially, and to the best of our knowledge, this was not been investigated before. (One might argue that the RWHE formulation seems to complicate the extrinsic calibration problem that was originally compared; however, we demonstrate that this is not a problem as shall be seen throughout the paper.) Third, such a formulation does not constrain the extrinsic calibration setup of the multi-sensor systems of a 2D lidar and camera pair to have an overlapping field of view. This allows for many possible calibration configurations of the underlying sensors. It is important to mention that this work is only concerned with the spatial extrinsic calibration of a 2D lidar–camera system, but not the temporal calibration. Readers interested in temporal calibration are advised to refer to the work of Furgale et al. [9].

The remainder of this paper is structured as follows. Section 2 provides an overview of existing approaches, recent work and contributions of this paper to the extrinsic calibration problem of a 2D lidar and camera system without overlap. Section 3 briefly describes the formulation of the robot–world hand–eye calibration problem. Section 4 first presents several configurations of 2D lidar and camera sensors, which we used in our experimentations, followed by presenting our proposed RWHE calibration procedure to extrinsically calibrate them. In Section 5, the experimental results are discussed and compared with one of the state-of-the-art published reports, namely the work of Zhang and Pless [6]. Lastly, Section 6 concludes the paper with final comments and future directions.

## 2. Recent Work and Contributions

There are many extrinsic calibration procedures published in the literature for calibrating multiple lidar units [10], multiple cameras [11,12], or other variations of the lidars and cameras. However, the majority of published calibration approaches focus on the extrinsic calibration of a camera and a lidar. Such extrinsic calibration is well studied in computer vision and robotics literature for both 2D and 3D lidars. Published work in calibration approaches of lidar–camera systems can be classified as follows:

- Targetless extrinsic calibration of lidar–camera sensors. Such approaches generally depend on finding correspondences between features (e.g., edges, lines, corners) extracted from both the lidar and camera systems and then minimizing the reprojection error associated with them. In this category, there are two types of research:
  - sufficient or partial overlap does exist between the sensors [13,14,15]; and   
  - no overlap [16].
- Target-based extrinsic calibration of lidar–camera sensors. Some examples of the calibration targets include a planar checkerboard pattern [6,7] or a right-angled triangular checkerboard [17] or a trirectangular trihedron [8] or an arbitrary trihedron [18] or a v-shaped calibration target [19] or others. In this category, there are two types of research:
  - sufficient overlap does exist between the field of views of the camera and lidar sensors [2,6,7,8,18,19,20,21,22,23,24,25,26,27,28]; and
  - no overlap [29].

Target-based extrinsic calibration with no overlap has received very little attention of research [29]. According to [29], this category includes two more categories. The first category is based on using a bridging sensor whose relative pose from both the camera and the lidar sensors can be calculated. The second category is based on using a reasonable assumption about specific geometric structures defined in the calibration environment. The calibration approach presented in this paper best fits in this latter category of non-overlapping target-based extrinsic calibration similar to what was discussed in [29]. In addition, the presented approach applies in the case of overlapping sensors similar to the work in [6].

In what follows, we briefly review some of the state-of-the-art approaches in extrinsic calibration of lidar–camera sensors. Afterward, we present our contributions to the state-of-the-art.

### 2.1. Targetless

Castorena et al. [15] proposed a joint automatic calibration and fusion approach for a 3D lidar and a camera via natural alignment of depth and intensity edges without requiring any calibration target. The approach jointly optimizes calibration parameters and fusion output using an appropriate cost function. This requires the sensors to have an overlapping field of view.

Pandey et al. [14] proposed a targetless calibration approach for calibrating 3D lidar and camera sensors that share a common field of view. The approach is based on using appearance of the environment by maximizing mutual information between sensor-measured surface intensities.

Napier et al. [16] calibrated a 2D push broom lidar to a camera and required no overlap or calibration target. The proposed approach relies on optimizing a correlation measure between lidar reflectivity and grayscale values from camera imagery acquired from natural scenes. However, the approach requires an accurate pose estimate from an inertial measurement unit (IMU) mounted on a moving platform.

### 2.2. Target-Based

Wasielewski and Strauss [20] proposed a calibration approach for a monochrome charge-coupled device (CCD) camera and a 2D lidar. They used a specific calibration pattern consisting of two intersected planes to match data provided by the camera and lidar sensors and therefore to identify the lidar–camera geometric transformation. The calibration parameters were estimated using nonlinear least squares. Similarly, Yu et al. [1] calibrated a lidar and a camera for data fusion using camera calibration toolbox for MATLAB  [30].

Geiger et al. [26] presented an online toolbox to fully automate camera-to-camera and camera-to-range calibration using a single image and 3D range scan shot. The approach uses a set of planar checkerboard calibration patterns, which are placed at various distances and orientations. The approach requires that the camera(s) and the 3D lidar sensors have a common field of view, which may or may not include all of the checkerboard patterns. Given one image or scan from each sensor, 3D range data is segmented into planar regions to identify possible checkerboard mounting positions. An exhaustive search is performed over all possible correspondences of pattern locations to find the optimal desired transformation estimates.

Gong et al. [18] proposed an approach for calibrating a system composed of a 3D lidar and a camera. They formulated the calibration problem as a nonlinear least squares in terms of geometric constraints associated with a trihedral object.

Khosravian et al. [28] proposed a Branch-and-Bound (BnB) approach for checkerboard extraction in the camera-lidar calibration problem. They formulated the checkerboard extraction as a combinatorial optimization problem with a defined objective function that relies on the underlying geometry of the camera-lidar setup. The BnB approach was used for optimizing the objective function with a tight upper bound to find the globally optimal inlier lidar points (i.e., the lidar scan points and their corresponding camera images). The approach requires that the calibration target is placed within the field of view of both the camera and the lidar sensors.

Zhang and Pless [6] proposed a practical implementation for the extrinsic calibration of a camera and 2D lidar sensors. The implementation requires that a planar calibration pattern is posed such that it is in the field of view of the camera and the lidar. The implementation also requires at least five planes input (five planar shots). Initially, a solution that minimizes an algebraic error is computed. Next, the computed solution is nonlinearly refined by minimizing a camera reprojection error. The implementation lacks a systematic study on the minimal solution of the original problem [31]. This implementation has been extended to extrinsically calibrate other kinds of lidars and cameras, and since then has become state of the art [31]. For example, a similar implementation for full 3D lidars is described in [32]. An efficient implementation of [6] through an online toolbox is given in [33,34].

The lack of the minimal solution issue in the Zhang and Pless implementation [6] motivated other studies to deal with this issue such as Vasconcelos et al. [7], Zhou [31], and Hu et al. [8]. For example, Vasconcelos et al., only using three planar shots, derived the minimal solution; however, it requires solving a sophisticated perspective-three-point (P3P) problem in dual 3D space with eight solutions, and might suffer from degeneration problems as discussed in [8]. Zhou’s study proposed an algebra method instead of geometric ones based on three plane-line correspondences to derive the minimal solution; however, multiple solutions and degeneration problems also exist as in the Vasconcelos’s method. The study in Hu et al. [8] derived the minimal solution only using a single shot of a trirectangular trihedron calibration object. The study avoided the degeneration problem found in Vasconcelos et al. and Zhou. It showed the ability to obtain a unique solution based on solving a simplified P3P and perspective-three-line (P3L) problems separately for the lidar and the camera poses, respectively. However, to uniquely determine the actual camera pose without scaling ambiguity from the formulated P3L problem, they needed some metric information from the scene, such as the actual lengths of two edges of the used trirectangular trihedron. Unfortunately, the availability of such information was not discussed further in the paper.

Bok et al. [29] proposed an approach for calibrating a camera and a 2D lidar, which requires no overlap between the camera-lidar sensors. The approach relies on an initial solution computed via singular value decomposition (SVD). The approach adopts a reasonable assumption about geometric structures in the calibration scene—either a plane or a line intersecting two planes. Then, the solution is refined via nonlinear optimization process to minimize Euclidean distance between the structures and the lidar data.

### 2.3. Our Contributions

Our contributions to the state of the art in extrinsic lidar–camera calibration are summarized as follows:

- The formulation of the extrinsic 2D lidar–camera calibration problem as a variant of the robot–world hand–eye calibration problem to support a non-overlapping field of view of the sensors.
- The computation of the transformation matrix B in the RWHE linear formulation AX=ZB, based on reasonable assumptions about geometric structure in the calibration environment.
- A novel approach for calibration to allow flexibility for sensor and calibration object placement.
- A calibration paradigm that involves a moving robotic platform and a stationary calibration object, which allows for automatic or semi-automatic robotic platform self-calibration.
- A demonstration of the proposed calibration approach on real sensors, including multiple configurations of lidars and cameras.
- Results were compared with a state-of-the-art method in lidar–camera calibration.

## 3. Robot–World Hand–Eye Calibration Problem

The RWHE calibration problem is modeled as the linear relationship AX=ZB, where X and Z are unknown calibration matrices composed of rotation and translation components. Calibration matrices (A,X,Z,andB) are represented by 4×4 homogeneous affine transformation matrices (HTMs), where the last row of each HTM is set as [0,0,0,1]. Each HTM matrix represents a distinct transformation function between two coordinate frames.

**Figure 1:**
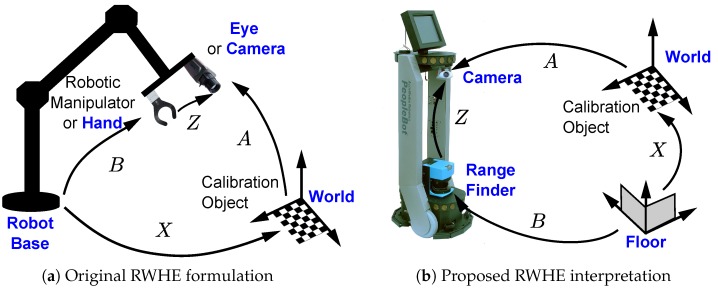
Hand–Eye Robot–World calibration formulation: (**a**) original; (**b**) proposed interpretation.

The original RWHE calibration formulation has four coordinate frames: world, camera, robot base and robot manipulator [5] as shown in Figure 1a. However, in this work, we assume having the following coordinate frames: world, camera, floor, and 2D range finder as shown in Figure 1b. The floor coordinate frame is an important element in our interpretation of the RWHE problem. The floor coordinate frame is assumed to be a stationary frame with respect to the world frame (or calibration object) and is detectable in the laser range finder frame. Subsequently, we define the (A,X,Z,andB) HTMs as follows. The transformation from the world coordinate frame to the camera coordinate frame is defined as A. The HTM A is assumed to be known, which is usually calculated using a camera calibration procedure such as Zhang’s method in [35], where the world coordinate frame is defined by a calibration object in the workspace. The transformation from the floor coordinate frame to the 2D range finder frame is B, and is assumed to be known or at the least easily computable. The transformation from the floor coordinate frame to the world frame is the unknown HTM X. Finally, the transformation from the 2D range finder coordinate frame to the camera coordinate frame is the desired unknown HTM Z.

In practice, many different positions of the 2D lidar and camera are used to generate many relationships $A_{i}X=ZB_{i}$, i∈[0,n−1] to get a single estimate of both X and Z, where *n* is the number of robot poses used for the calibration.

Many different approaches exist in the literature for estimating X and Z, including linear and iterative methods such as the work of [36,37,38]. An accurate approach to robot–world hand–eye calibration was proposed in [39] based on the Euler parameterizations for rotations, which was recently extended to include other parameterizations as well (axis-angle and quaternions) [40]. Due to the high accuracy of this proposed approach [39,40], it has been adopted in this paper for estimating the extrinsic calibration parameters (Z) as will be discussed in the next section. Specifically, we used the “Euler parameterization I” method from [39] or what was then named as “c1, Euler angles, simultaneous” in [40]. Furthermore, we used the open source code accompany to [40], available at [41], for the implementation of the selected RWHE method. The open source code includes other methods or parameterizations of the rotational angles (angle-axis representation or quaternion), which we incorporated in our implementation as well. However, for the datasets that we recorded, Euler parameterization I comparably gave the best estimated results without singularity issues, hence it was selected in this paper. In the event of singularity issues, other parameterizations can be considered with little impact on our proposed approach.

## 4. The Proposed Calibration Procedure

The proposed RWHE calibration formulation consists of the following steps:

1. Select a configuration that relates the lidar–camera sensors together.
2. Define the calibration environment with respect to the RWHE coordinate frames (world, camera, floor, and 2D range finder) and the transformation matrices (A,B,X,Z).
3. Compute the transformation matrices A and B.
4. Apply the selected RWHE Euler parameterization I method to estimate the desired extrinsic calibration parameters Z.
5. Verify the accuracy of the estimated extrinsic calibration results.

In what follows, the above steps are discussed in detail.

### 4.1. Camera–Lidar Configurations

**Figure 2:**
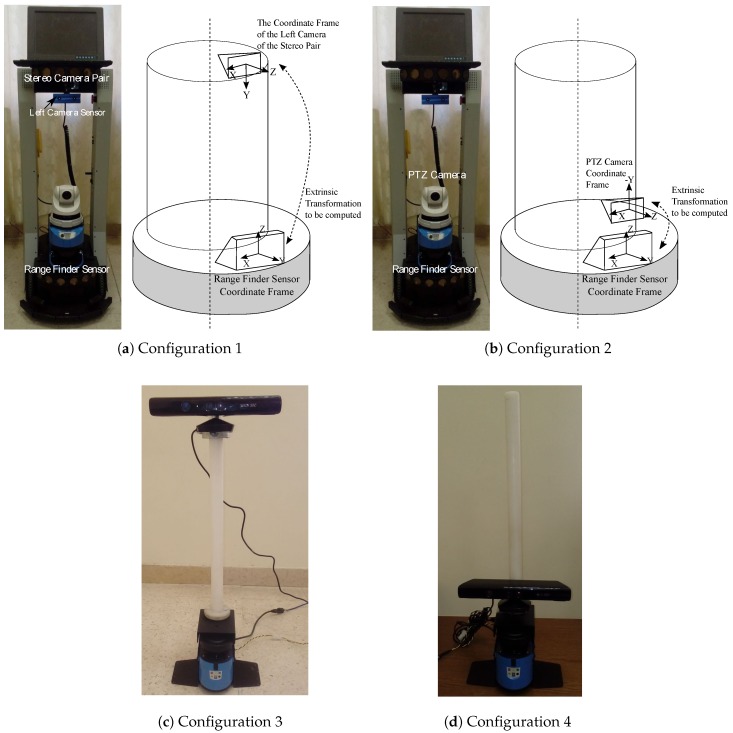
Four configurations of lidar–camera sensors: (**a**) SICK LMS-500 2D lidar (Minneapolis, MN, USA) and a mobileRanger C3D stereo camera (Focus Robotics, Hudson, NH, USA) rigidly attached to a PeopleBot robotic platform; (**b**) SICK LMS-500 2D lidar and AXIS 214 PTZ network camera (Axis Communications, Lund, Sweden) rigidly attached to a PeopleBot robotic platform; (**c**) SICK LMS-100 2D lidar and a Kinect Xbox 360 camera rigidly attached at a height similar to a mobile robot; (**d**) same configuration as of (**c**), but the camera is mounted at a shorter vertical distance from the lidar.

To compute the calibration parameters, we considered the following camera–lidar configurations:

- Configuration 1 consists of a left camera sensor of a stereo camera pair and a 2D lidar attached to a PeopleBot robotic platform (Adept MobileRobots LLC., Amherst, NH, USA), as shown in Figure 2a. The combination of the lidar–camera sensors in this configuration is assumed to represent a non-overlapping field of view of the sensors (or in other words the lidar scans need not be on the calibration object imaged by the camera sensor).
- Configuration 2 consists of a pan-tilt-zoom (PTZ) camera sensor and a 2D lidar attached to the PeopleBot robot, as shown in Figure 2b. The combination of the lidar–camera sensors in this configuration is assumed to represent an overlapping field of view of the sensors (the lidar scans are on the calibration object imaged by the camera sensor). The goal of configurations 1 and 2 is to compute the extrinsic transformation between the 2D lidar sensor and each of the left camera sensor of the stereo pair and the PTZ camera. Such transformations are required for sensor data fusion of different applications such as simultaneous localization and mapping (SLAM) [42,43], place recognition and robot self-localization [44], and others.
- Configuration 3 consists of an Xbox 360 Kinect V1 camera sensor (Microsoft, Washington, DC, USA) and a 2D lidar sensor attached together. The vertical distance between the Kinect camera and the 2D lidar simulates that of an indoor mobile robot. See Figure 2c.
- Configuration 4 is similar to configuration 3, except that the vertical distance between the camera and the 2D lidar sensor is shorter as depicted in Figure 2d. In configurations 3 and 4, no robot is involved, but the problem remains in the context of mobile robots or autonomous systems in that two sensors need to be calibrated together in order to accomplish a task. Although the camera that we used in configurations 3 and 4 is the Xbox 360 Kinect V1 sensor, we are only using the RGB data (the depth data was not used).

All of the configurations above were used in our experimentations as shall be discussed in Section 5.

### 4.2. Calibration Environment

To apply the RWHE formulation AX=ZB on the underlying extrinsic calibration problem, we need to further discuss the involved coordinate frames introduced in Section 3.

**Figure 3:**
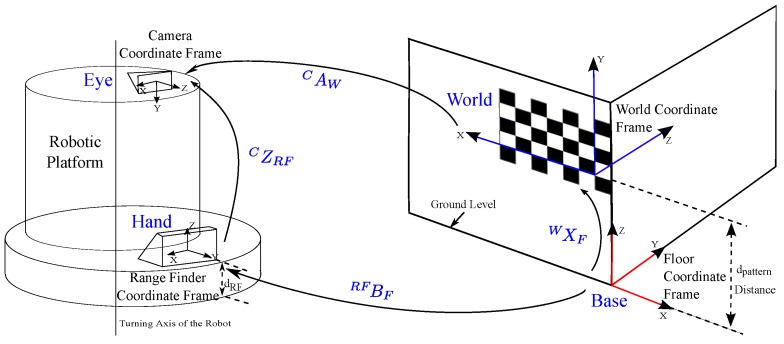
The calibration environment for configuration 1.

From the standard RWHE calibration problem, there is the world coordinate frame that is defined by a stationary calibration object. In this work, we decided to use a planar chessboard pattern. There is also the floor coordinate frame that serves as the reference coordinate frame for the lidar sensor. As mentioned before, the main requirement on this floor frame is to be a stationary with respect to the calibration object and detectable by the lidar sensor. A corner of a wall in the calibration workspace was chosen for this floor frame, using the assumption that the wall planes are orthogonal. This is shown in Figure 3 for configuration 1. We should mention that Figure 3 also serves as the calibration environment for the rest of the configurations in Figure 2.

Additionally, the camera and the 2D lidar have their own coordinate frames, giving a total of four coordinate frames that are related by linear transformations (A,B,X,Z). Consequently, the relationships of the four coordinate frames can be modeled using the linear relationships of the RWHE calibration problem that is given by Equation (1):

(1) AX=ZB.

Substituting in the superscripts and subscripts for clarity and completeness (*C* = camera, *W* = world calibration pattern, *F* = floor coordinate frame, RF = lidar or range finder coordinate frame), we get the following equation:

(2) $${}^{C}A_{W}{}^{W}X_{F}={}^{C}Z_{RF}{}^{RF}B_{F}.$$

The different transformations shown in Equation (2) are depicted in Figure 3, which show how the extrinsic calibration problem of a 2D lidar–camera system is now a valid variant of the general RWHE calibration problem. Our main objective is to estimate the ${}^{C}Z_{RF}$ matrix between the lidar and camera frames in addition to the ${}^{W}X_{F}$ matrix.

We can solve the formulated calibration problem in Equation (2) for ${}^{W}X_{F}$ and ${}^{C}Z_{RF}$ using the established methods for the robot–world hand–eye [39,40]. This requires computing the transformation matrices ${}^{C}A_{W}$ and ${}^{RF}B_{F}$. ${}^{C}A_{W}$ is assumed to be computable using a camera calibration procedure such as Zhang [35]. ${}^{RF}B_{F}$ is also assumed to be computable, though with extensive efforts. To compute ${}^{RF}B_{F}$ we used the 2D range data obtained from the lidar sensor and made several assumptions. In what follows, we describe our procedure and assumptions for computing ${}^{RF}B_{F}$.

### 4.3. The Computation of the ${}^{RF}B_{F}$ Matrix

Originally, we assumed to have a corner of the wall to define the floor coordinate frame. The transformation matrix ${}^{RF}B_{F}$ is not fixed because it varies according to each pose of the 2D lidar sensor with respect to the floor coordinate frame. Hence, we relied on the 2D lidar point measurements in our computation of the ${}^{RF}B_{F}$ matrix. Using the mathematical notation, a 2D lidar point measurement is usually expressed in polar coordinates (ρ, θ). ρ is the measured radial distance of the lidar beam at an angle θ in the *x*−*y* plane of the lidar frame at a certain vertical distance from the ground level ($d_{RF}$) as defined by the floor coordinate frame’s *z*-axis. For example, using the coordinate frames in Figure 3, $d_{RF}$ distance represents the perpendicular distance between the origin of the lidar sensor and the ground. The $d_{RF}$ distance is assumed to either be manually measured or given in the robotic platform’s manual that comes already equipped with a lidar sensor. Thus, in terms of the Cartesian coordinates, a lidar point measurement (x,y,z) at certain angular resolution can be expressed in the lidar frame as described below:

(3) $$\begin{array}{c} x=\rho \times cos(\theta ),\\ y=\rho \times sin(\theta ),\\ z=0. \end{array}$$

**Figure 4:**
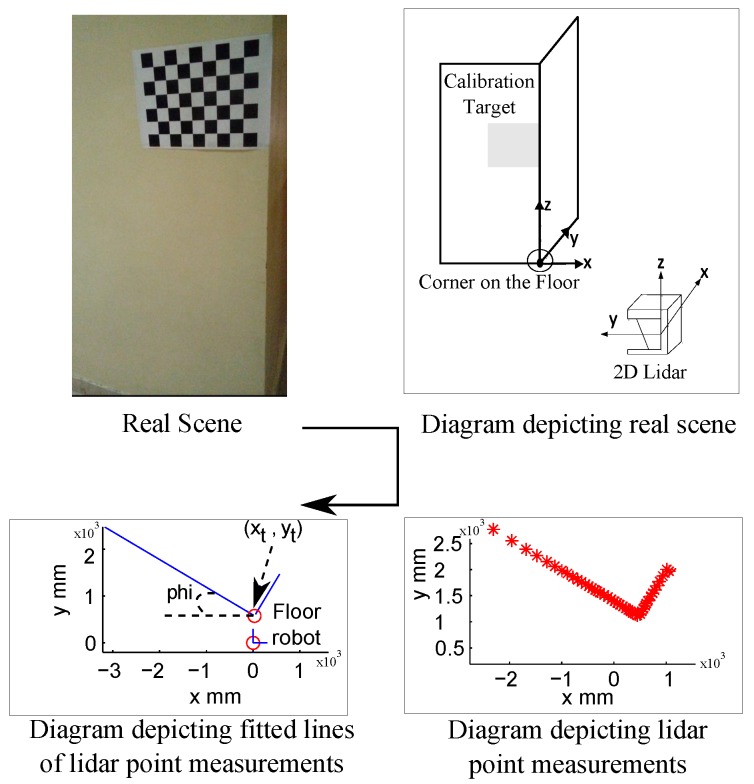
Steps to estimate the ${}^{RF}B_{F}$ matrix.

During the calibration procedure, we assume that the calibration data is being recorded during stop and go mode of the robotic platform against the calibration object as shown in Figure 3. The calibration data contains an image of the calibration object and a lidar scan at each position of the robot platform (or lidar–camera pair) as recorded by the camera and lidar sensors. Each recorded lidar scan consists of a large number of lidar point measurements. The lidar points from each lidar scan are supplied to a linear least squares line fitting procedure to generate a 2D line map. This map is used to compute the ${}^{RF}B_{F}$ matrix after locating the origin of the floor frame with respect to the origin of the lidar frame (i.e., (0,0) location on the map). Locating the origin of the floor frame within the map allows computation of three translational components ($x_{t}$, $y_{t}$ and $d_{RF}$) and one rotational component (ϕ) between the origins of the floor and lidar frames of the ${}^{RF}B_{F}$ matrix. This provides the transformation ${}^{RF}B_{F}$ under the assumption that the lidar scans are all parallel to the floor *x*-*y* plane (i.e., the roll and pitch angles of the lidar are zeros). We understand that this assumption might be considered a limitation of the proposed work; however, this will mainly affect how the rotational components of the ${}^{RF}B_{F}$ matrix are computed. We believe that the presented work with such an assumption can be tolerated as to just prove the main idea of applying the RWHE formulation for the extrinsic lidar–camera calibration with no overlapping field of views of the sensors. The computed ${}^{RF}B_{F}$ matrix is expressed as given in Equation (4). Figure 4 summarizes the steps taken to compute the ${}^{RF}B_{F}$ matrix for one pose of the lidar–camera pair:

(4) $${}^{RF}B_{F}=\left[\begin{array}{cccc} cos(\phi ) & -sin(\phi ) & 0 & x_{t}\\ sin(\phi ) & cos(\phi ) & 0 & y_{t}\\ 0 & 0 & 1 & -d_{RF}\\ 0 & 0 & 0 & 1 \end{array}\right].$$

### 4.4. Solving the Extrinsic Calibration Parameters

After computing the transformations ${}^{C}A_{W}$ and ${}^{RF}B_{F}$, the ${}^{W}X_{F}$ and ${}^{C}Z_{RF}$ transformations can be estimated using the Euler parameterization I method [39]. In Euler parameterization I, the cost function to be minimized is given in Equation (5), where ${}^{C}A_{W,i}$ represents the *i*th camera pose. The transformations ${}^{W}X_{F}$ and ${}^{C}Z_{RF}$ are decomposed into rotation and translation components as shown in Equation (6). Therefore, in the minimization process, we have a function of 12 variables: three Euler angles and three translation components for each of the ${}^{W}X_{F}$ and ${}^{C}Z_{RF}$ matrices:

(5) $$c_{1}=\sum_{i=0}^{n-1}\left|\right|{}^{C}A_{W,i}{}^{W}X_{F}-{}^{C}Z_{RF}{}^{RF}B_{F,i}\left|\right|{}^{2},$$

(6) $$c_{1}=\underset{i=0}{\sum^{n-1}}{∥A_{i}\left[\begin{array}{cc} R_{X}\left(\theta_{a},\theta_{b},\theta_{c}\right) & \begin{array}{c} t_{X0}\\ t_{X1}\\ t_{X2} \end{array}\\ \vec{0} & 1 \end{array}\right]-\left[\begin{array}{cc} R_{Z}\left(\theta_{d},\theta_{e},\theta_{f}\right) & \begin{array}{c} t_{Z0}\\ t_{Z1}\\ t_{Z2} \end{array}\\ \vec{0} & 1 \end{array}\right]B_{i}∥}^{2}.$$

The overall minimization problem is shown in Equation (7):

(7) $$\left\{{\hat{\theta }}_{a},{\hat{\theta }}_{b},{\hat{\theta }}_{c},{\hat{t}}_{X0},{\hat{t}}_{X1},{\hat{t}}_{X2},{\hat{\theta }}_{d},{\hat{\theta }}_{e},{\hat{\theta }}_{f},{\hat{t}}_{Z0},{\hat{t}}_{Z1},{\hat{t}}_{Z2}\right\}=\underset{\begin{array}{c} \theta_{a},\theta_{b},\theta_{c},t_{X0},t_{X1},t_{X2}\\ \theta_{d},\theta_{e},\theta_{f},t_{Z0},t_{Z1},t_{Z2} \end{array}}{\mathrm{arg min}}c_{1}.$$

An approximate solution to Equation (7) is found using an L2 norm with the Levenberg–Marquardt method for nonlinear least squares, as provided by the implementation levmar [45]. (The initial solutions of $\theta_{a},\theta_{b},\theta_{c},\theta_{d},\theta_{e},\theta_{f}$ are set to zeros such that the corresponding rotational matrices are identity, and similarly the translation components are set to zero. It is important to mention that for the selected RWHE method, as quoted from [40], “various different initial solutions were tested, and there was small or negligible difference in the solution quality versus using an identity matrix for rotation matrices and translation component with all elements zero. For this reason, we conclude that for the experiments ... for the first class of methods is not sensitive to initial solutions”. Therefore, the reader should notice that, with initial solutions as set above, the proposed extrinsic calibration approach will still converge even if the orientations of the camera and the lidar are highly not aligned (i.e., a rotation that is not being closed to the identity) as well as a translation that is not fairly easy to estimate.) Then, substituting in the estimated parameters gives ${}^{W}X_{F}$ and ${}^{C}Z_{RF}$ in approximate forms ${}^{W}{\hat{X}}_{F}$ and ${}^{C}{\hat{Z}}_{RF}$ as shown in Equations (8) and (9):

(8) $$\begin{array}{cc} {}^{W}{\hat{X}}_{F}= & \left[\begin{array}{cc} R_{X}\left({\hat{\theta }}_{a},{\hat{\theta }}_{b},{\hat{\theta }}_{c}\right) & \begin{array}{c} {\hat{t}}_{X0}\\ {\hat{t}}_{X1}\\ {\hat{t}}_{X2} \end{array}\\ \vec{0} & 1 \end{array}\right], \end{array}$$

(9) $$\begin{array}{cc} {}^{C}{\hat{Z}}_{RF}= & \left[\begin{array}{cc} R_{Z}\left({\hat{\theta }}_{d},{\hat{\theta }}_{e},{\hat{\theta }}_{f}\right) & \begin{array}{c} {\hat{t}}_{Z0}\\ {\hat{t}}_{Z1}\\ {\hat{t}}_{Z2} \end{array}\\ \vec{0} & 1 \end{array}\right]. \end{array}$$

**Figure 5:**
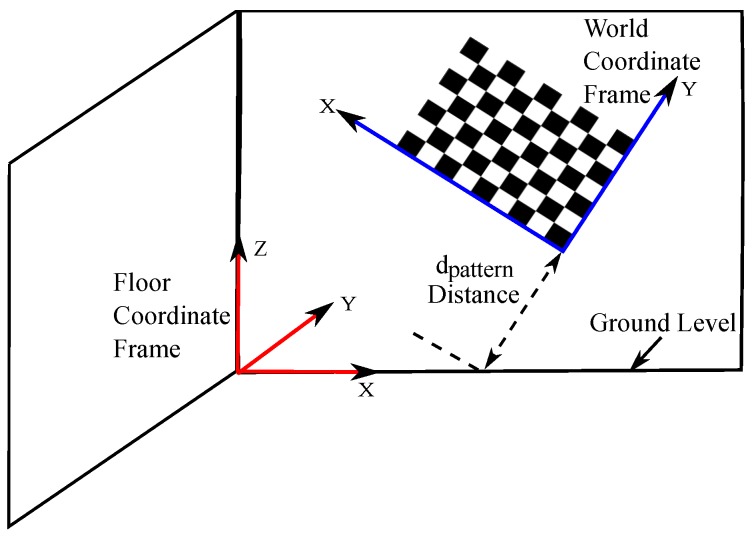
Schematic view of possible orientation of the world coordinate system.

The transformations ${}^{W}{\hat{X}}_{F}$ and ${}^{C}{\hat{Z}}_{RF}$ will have an ambiguity in the translation components. This is explained as follows. The reader should note that since the used lidar is only a 2D sensor, the distance between the world coordinate frame and the floor coordinate frame is not constrained by Equation (5). Moreover, in the calibration procedure, when we recorded our calibration datasets, the calibration object was assumed to be stationary with a moving robotic platform against it. Hence, considering the sensors attached to a heavy weight robot platform, similar to the ∼25 Kg PeopleBot platform in our configurations 1 and 2, we will have a problem in our recorded datasets. The problem is that the moving robot platform will not have tilting poses during the calibration procedure. This results in that the recorded calibration datasets lack any excitation in the roll and pitch angles, which in turn renders the mounting height of the camera unobservable and accordingly an ambiguity in the translational components of the estimated extrinsic parameters. However, we can create a constraint and solve this problem by manually measuring a defined distance that we call $d_{pattern}$. $d_{pattern}$ distance is defined as the distance (in terms of the world coordinate frame’s *y* axis) from the (0,0,0) location of the calibration object to the ground plane. However, other configurations of the world frame are possible, such as in Figure 5. In this case, to find $d_{pattern}$ distance, some trigonometry is needed in terms of measuring the perpendicular distance from the origin of the world frame along the *y*-axis to the ground.

When the value of $d_{pattern}$ is known, we can specify that the *y* translation component of ${}^{W}X_{F}$ is $-d_{pattern}$ as shown in Equation (10):

(10) $$\begin{array}{c} {}^{W}{\hat{X}}_{F}=\left[\begin{array}{cc} R_{X}\left({\hat{\theta }}_{a},{\hat{\theta }}_{b},{\hat{\theta }}_{c}\right) & \begin{array}{c} {\hat{t}}_{X0}\\ -d_{pattern}\\ {\hat{t}}_{X2} \end{array}\\ \vec{0} & 1 \end{array}\right]. \end{array}$$

While it is possible to adjust ${}^{W}{\hat{X}}_{F}$ with a constant such that the translational ambiguity is resolved, in order to reconcile ${}^{C}{\hat{Z}}_{RF}$ to that change, another Levenberg–Marquardt minimization procedure is recommended to be performed.

Consequently, we minimize the function $c_{1}$ in Equation (6) via another application of the Levenberg–Marquardt method, but this time with one less parameter. This is because the *y* component of the translation in ${}^{W}{\hat{X}}_{F}$ will remain constant as $-d_{pattern}$. The minimization problem is shown in Equation (11); the initial solution given to the Levenberg–Marquardt method is ${}^{W}{\hat{X}}_{F}$ and ${}^{C}{\hat{Z}}_{RF}$. The new approximate solutions ${}^{W}{\hat{\hat{X}}}_{F}$ and ${}^{C}{\hat{\hat{Z}}}_{RF}$ are produced by substituting the estimated parameters as in Equations (8) and (9) and ${\hat{\hat{t}}}_{X1}=-d_{pattern}$:

(11) $$\left\{{\hat{\hat{\theta }}}_{a},{\hat{\hat{\theta }}}_{b},{\hat{\hat{\theta }}}_{c},{\hat{\hat{t}}}_{X0},{\hat{\hat{t}}}_{X2}{\hat{\hat{\theta }}}_{d},{\hat{\hat{\theta }}}_{e},{\hat{\hat{\theta }}}_{f}{\hat{\hat{t}}}_{Z0},{\hat{\hat{t}}}_{Z1},{\hat{\hat{t}}}_{Z2}\right\}=\underset{\begin{array}{c} \theta_{a},\theta_{b},\theta_{c},t_{X0},t_{X2}\\ \theta_{d},\theta_{e},\theta_{f},t_{Z0},t_{Z1},t_{Z2} \end{array}}{\mathrm{arg min}}c_{1}.$$

Algorithm 1 summarizes the process of estimating the transformations ${}^{W}{\hat{\hat{X}}}_{F}$ and ${}^{C}{\hat{\hat{Z}}}_{RF}$. It is recommended that the order in Algorithm 1 of lines 3, 5, and 6 is maintained. The reason is that the search for an approximate local minimum gets stuck in poorer quality estimates of ${}^{W}X_{F}$ and ${}^{C}Z_{RF}$ if this order is not followed. Of course, this behavior is method and dataset dependent. In addition, it may not be true in all situations, but, through experimentation, it was found true with the datasets that we used in this work.

**Algorithm 1** Perform calibration of two sensors
1:Compute ${}^{C}A_{W,i}$ for each pose *i*.2:Compute ${}^{RF}B_{F,i}$ for each pose *i*.3:Compute solution to Equation (7) using 12 parameters, using zeros as initial solutions for all parameters. This solution is ${}^{W}{\hat{X}}_{F}$ and ${}^{C}{\hat{Z}}_{RF}$.4:Manually measure the $d_{pattern}$ distance.5:Set the *y* translational component of ${}^{W}{\hat{X}}_{F}$ to $-d_{pattern}$ as in Equation (10).6:Compute ${}^{W}{\hat{\hat{X}}}_{F}$ and ${}^{C}{\hat{\hat{Z}}}_{RF}$, which are the approximate solution to Equation (11) using 11 parameters, where ${}^{W}{\hat{X}}_{F}$ and ${}^{C}{\hat{Z}}_{RF}$ (from line 3) serve as the initial solution.


### 4.5. Verifying Accuracy

With the extrinsic calibration results ${}^{C}{\hat{\hat{Z}}}_{RF}$ and ${}^{W}{\hat{\hat{X}}}_{F}$ estimated using Algorithm 1, the laser data from the 2D lidar can be projected onto the imaging plane of the camera sensor. This will be an important step to check the accuracy of the estimated results. To this end, we next discuss how a given lidar point measurement is projected onto the camera sensor plane.

To project a homogeneous point ${\vec{X}}_{i}$ defined with respect to the lidar coordinate frame (see Equation (3)) onto a point ${\vec{x}}_{i}$ in the image plane of a camera sensor, we can use the following forward projection relationship:

(12) $${\vec{x}}_{i}=K{\hat{\hat{Z}}}_{3\times 4}{\vec{X}}_{i},$$

where ${\vec{x}}_{i}$ is composed of a (3×1) homogeneous vector, ${\vec{X}}_{i}$ is composed of a (4×1) homogeneous vector (the third *z* component is zero), $K_{3\times 3}$ is the intrinsic camera calibration matrix, and ${\hat{\hat{Z}}}_{3\times 4}$ is ${}^{C}{\hat{\hat{Z}}}_{RF}$ without the last row (i.e., [0,0,0,1]). The relationship in Equation (12) assumes either zero radial and tangential distortion or that such distortion has already been removed.

Similarly, with the estimated calibration results (${}^{C}{\hat{\hat{Z}}}_{RF}$ and ${}^{W}\hat{\hat{X_{F}}}$), the world calibration points on the calibration pattern can be projected onto the imaging plane using a newly estimated A matrix that we refer to as $A_{new}={}^{C}{\hat{\hat{Z}}}_{RF}\;{}^{RF}B_{F}\;{}^{W}{\hat{\hat{X}}}_{F}^{-1}$ matrix. This is done using the forward projection equation as follows:

(13) $${\vec{x}}_{i}=KA_{new_{3\times 4}}{\vec{X}}_{i},$$

where ${\vec{x}}_{i}$ is again composed of a (3×1) homogeneous vector, ${\vec{X}}_{i}$ is composed of a (4×1) homogeneous vector in the world coordinate frame, $K_{3\times 3}$ is the intrinsic camera calibration matrix, and $A_{new_{3\times 4}}$ is $A_{new}$ without the last row. The relationship in Equation (13) also assumes either zero radial and tangential distortion or that such distortion has already been removed.

Subsequently, we could check the accuracy of the estimated calibration results ${}^{C}{\hat{\hat{Z}}}_{RF}$ and ${}^{W}{\hat{\hat{X}}}_{F}$, by computing the reprojection root mean squared error (*rrmse*). The *rrmse* is computed between projected world calibration points (i.e., the grid points of the calibration object) to the image plane using the original A matrix and the same world calibration points projected using the $A_{\mathrm{new}}$ matrix. This *rrmse* computation is given in Equation (14):

(14) $$rrmse=\sqrt{\frac{1}{m}\sum_{i}^{m}{∥{\vec{x}}_{i,new}-{\vec{x}}_{i}∥}^{2}},$$

where *i* is an image point index, *m* total number of world projected calibration points, ${\vec{x}}_{i,new}\;=\;KA_{new_{3\times 4}}{\vec{X}}_{i}$, and ${\vec{x}}_{i}\;=\;KA_{3\times 4}{\vec{X}}_{i}$.

## 5. Experimental Results

This section presents the experimental extrinsic calibration results for the four configurations shown in Figure 2. For configurations 1 and 2, in Figure 2a,b, a SICK-LMS 500 lidar is used. This lidar was set to an angular resolution of $0.5^{\circ }$ and $180^{\circ }$ angular field of view. For configurations 3 and 4, in Figure 2c,d, a SICK-LMS 100 is used with an angular resolution of $0.5^{\circ }$ and $270^{\circ }$ angular field of view.

In all configurations (1–4), we assume valid and known intrinsic calibration parameters of the cameras. We also assume a pin-hole camera model for the cameras with radial and tangential lens distortion as described in [35]. (It is assumed that, for configurations 1 and 2 in Figure 2, the individual intrinsic camera calibration and the remaining extrinsic transformation, say between the left and right camera or between the left camera and the PTZ camera sensors, to be computed using the standard stereo camera calibration [30,46] when needed.) All of the results shown in this paper were generated on a workstation with two quad-core processors and 8 GB of RAM. The camera calibration was carried out using the open source computer vision library (i.e., OpenCV’s camera calibration functions) [46], where the calibration object does not have to be moved. The calibration object used in all configurations was a 9×7 checkerboard, where the size of a checker square was 40 mm × 40 mm.

The number of calibration images and lidar scans used in configurations 1 and 2 was 18, while 15 and 12 were used for configurations 3 and 4, respectively. The number of poses reported above for all of the configurations has no link to the accuracy of the reported results, but rather an indication of the actual poses that was recorded and used to generate the estimated calibration results.

**Figure 6:**
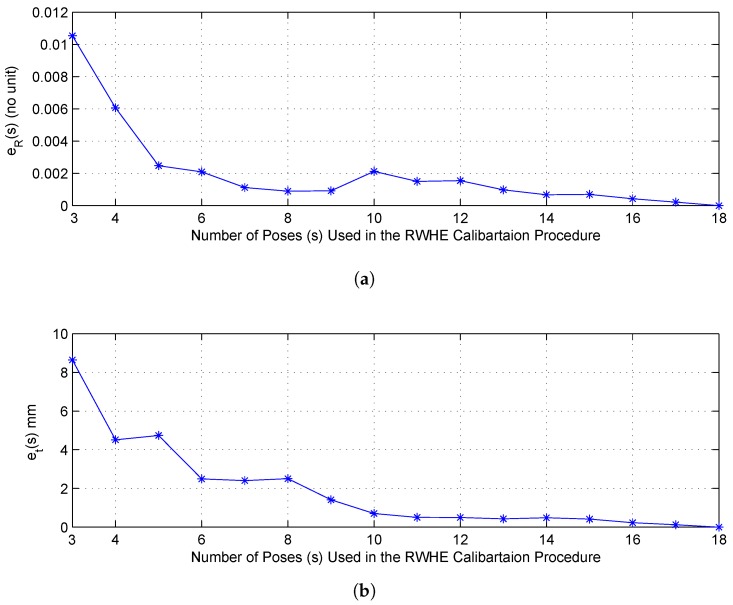
The accuracy of the extrinsic camera calibration parameters as a function of the number of poses used in the RWHE calibration procedure for configuration 2: **(a)** rotation error of the estimated Z matrix; **(b)** translation error of the estimated Z matrix.

It should be mentioned that when more poses (i.e., calibration images and lidar scans) are used during the calibration procedure, then more robust estimation of the intended calibration parameters are obtained. However, the observed changes in accuracy were not significant when the number of poses is larger than 10 in terms of both of the rotational and translational components. This was actually deduced from Figure 6 for configuration 2 (other configurations have a similar trend). To generate such a figure and stated observation above, we set the minimum number of poses, to be used in the RWHE calibration procedure, to 3 (the minimum possible) and 18 for the maximum number of poses (i.e., all of the recorded poses). Then, we randomly generated an array of indices from the range of all of the 18 poses ($n_{poses}$). The size (*s*) of the generated array is set to vary from 3 to 17 with an increment of one. The poses correspond to each of the generated array of *s* indices are fed to the RWHE calibration procedure to estimate the desired calibration parameters X and Z. To avoid being biased toward certain set of poses and thus to a particular set of estimated results, this process of randomly selecting the set of *s* poses from all of the available poses and estimating the calibration parameters is iterated several times (17 trials to be exact, which can be changed). Then, from all of the iterated experiments, the estimated calibration results are averaged over the total number of trials ($n_{trials}=17$). Afterwards, using all of the 18 poses, the calibration parameters are estimated. Particularly, we are interested in the Z matrix that is composed of the rotation matrix $R_{Z}$ or its Rodrigues vector representation that we denote ${\mathrm{Rod}}_{Z}$ and the translation vector $t_{Z}$. We are also interested in how much the accuracy of the estimated parameters get affected when using less number of posses (*s*) with respect to using the full set of poses. Consequently, using the L2 norm, we estimated the rotational errors ($e_{R}$) and translational errors ($e_{t}$), as a function of the number of poses used in the calibration procedure (*s*), which are shown below in Equation (15) and graphically plotted in Figure 6:

(15) $$\begin{array}{c} Average\left({\mathrm{Rod}}_{Z}\left(s\right)\right)=\frac{1}{n_{trials}}\sum_{k=1}^{n_{trials}}\left({\mathrm{Rod}}_{Z}\left(s,k\right)\right)\\ Average\left(t_{Z}\left(s\right)\right)=\frac{1}{n_{trials}}\sum_{k=1}^{n_{trials}}\left(t_{Z}\left(s,k\right)\right)\\ e_{R}\left(s\right)=∥Average\left({\mathrm{Rod}}_{Z}\left(s\right)\right)-{\mathrm{Rod}}_{Z}\left(n_{poses}\right)∥\\ e_{t}\left(s\right)=∥Average\left(t_{Z}\left(s\right)\right)-t_{Z}\left(n_{poses}\right)∥, \end{array}$$

where s∈3,4,…,17, $n_{trials}=17$, and $n_{poses}=18$.

From Figure 6 and related experiments discussed above, we can draw several observations.

- Using few poses produced calibration results comparable to that of large number of poses.
- Comparing three poses with 18 poses, the averaged translational errors were just 8 mm and the averaged rotation errors were just 0.0105 indicating that the presented RWHE calibration method works fine with the three poses case (i.e., minimum number of possible poses).
- The most important poses that would mostly affect the accuracy of the results are those poses that are very close to the calibration pattern, although this was not shown in Figure 6, as we are taking the average of the randomly selected poses over many trials. This observation was actually discussed in [29], and as such no experimental results about this study were presented and analyzed further in this paper.

Table 1 lists the manually measured distances $d_{RF}$ and $d_{pattern}$ for the Configurations 1–4.

Throughout all of the experiments, once the A and B matrices are determined, the time to estimate the $\hat{\hat{X}}$ and $\hat{\hat{Z}}$ transformations is usually less than a minute. Practically speaking, the exact time will depend on the size of the calibration data supplied to the calibration procedure. Further insight on the timing performance of the employed RWHE calibration procedure is provided in [40].

For the configurations in Figure 2, configuration 2 allows for overlapping filed of views of the sensors as done in Zhang and Pless method [6]. Hence, it was easier to compare this configuration with the work in [6].

The rest of the section is organized as follows. First, the calibration results (i.e., the estimated X and Z transformations) are presented for configurations 1 and 3, when the calibration pattern is not in the field of view of both the lidar and camera sensors (Section 5.1). Then, the results are discussed for configurations 2 and 4, when the lidar and camera sensors view the calibration pattern (Section 5.2). The comparison with the Zhang and Pless method [6] is presented in Section 5.3. The uniqueness of the proposed calibration approach is highlighted in Section 5.4.

### 5.1. Calibration Results for Configurations 1 and 3

To estimate the calibration parameters for configurations 1 and 3, we used the corresponding measured $d_{pattern}$ and $d_{RF}$ values provided in Table 1. The accuracy of the obtained results is calculated using Equation (14).

**Figure 7:**
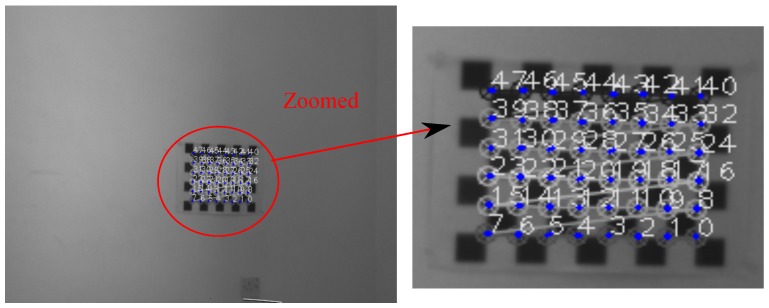
Configuration 1: Reprojection error based on the estimated X and Z matrices. Blue lines indicate the amount of the reprojection error (original image resolution 752×480).

**Figure 8:**
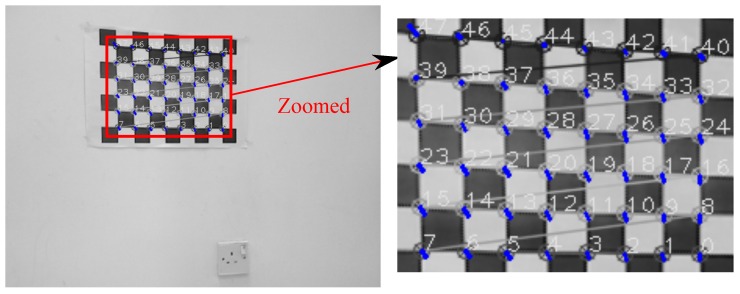
Configuration 3: Reprojection error based on the estimated X and Z matrices. Blue lines indicate the amount of the reprojection error (original image resolution 640×480).

Figure 7 and Figure 8 show projection of world calibration points (grid points) to one selected calibration image in configurations 1 and 3. The error difference between the location of the point in images (based on the original A matrix) and the reprojected point (based on the $A_{new}=ZBX^{-1}$ matrix) is shown by a blue line. The *rrmse* error values are (in pixels); 3.77658 and 5.17108 for configurations 1 and 3, respectively. The error values imply high accuracy in the estimated calibration results.

**Figure 9:**
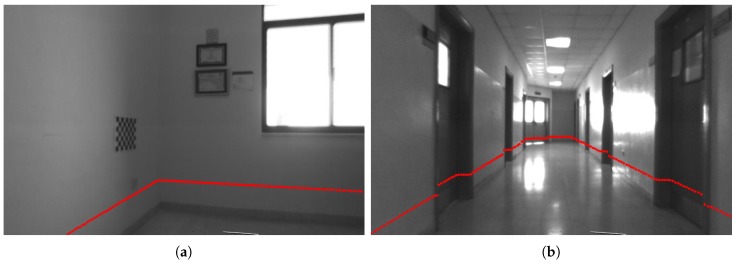
Configuration 1: Projection of lidar data to two test images using the estimated Z matrix (points are shown in red color): **(a)** first test image; **(b)** second test image.

**Figure 10:**
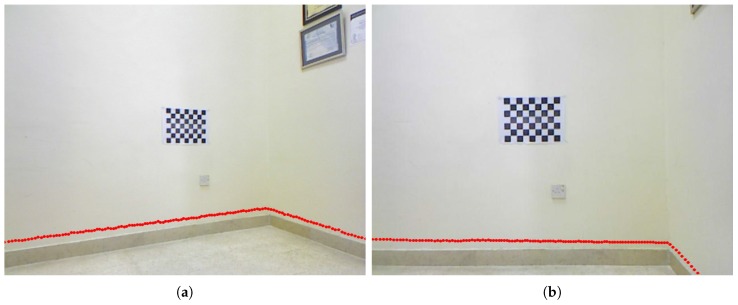
Configuration 3: Projection of the lidar data to two test images using the estimated Z matrix (points are shown in red color): **(a)** first test image; **(b)** second test image.

Figure 9 and Figure 10 show forward projection of lidar point measurements to two selected test images for configurations 1 and 3, using the corresponding estimated Z matrix. It should be noted that no ground truth of the estimated calibration parameters is available. With visual inspection, the lidar points are correctly projected to the image plane, in configurations 1 and 3, at about the same height of the corresponding lidar from the ground level based on the $d_{RF}$ distance. The verification was manually done by measuring the distances of where the lidar points projected on the wall plane(s) or on the calibration object (see next subsection), and then comparing that to the reported $d_{RF}$ values in Table 1. The measured distances of the projected lidar scans to the ground plane were all very close to the $d_{RF}$ values in Table 1 within 1–5% of an error. While this error is small, it is believed that the error sources might be due to uncertainties in the estimated intrinsic camera parameters, measured distances, lidar point measurements, and the computed A and B matrices. This is consistent with the assumption that the lidar scans are parallel to the ground plane, and shows high accuracy in the estimated calibration results. Similar verification was done for the rest of the configurations.

### 5.2. Calibration Results for Configurations 2 and 4

**Figure 11:**
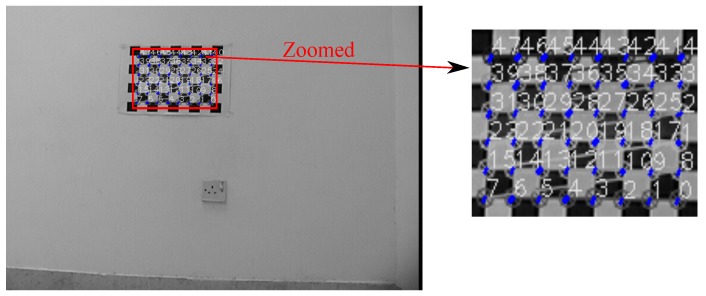
Configuration 2: Reprojection error based on the estimated X and Z matrices. Blue lines indicate the amount of the reprojection error (original image resolution 704×480).

**Figure 12:**
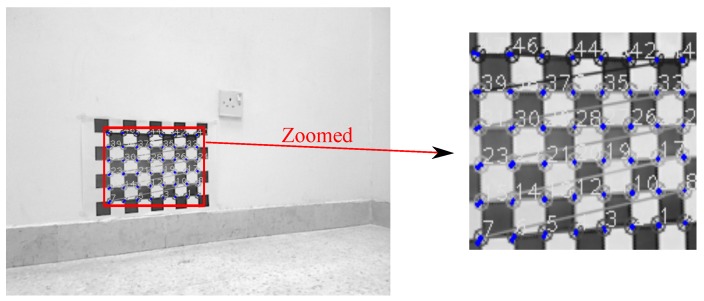
Configuration 4: Reprojection error based on the estimated X and Z matrices. Blue lines indicate the amount of the reprojection error (original image resolution 640×480).

**Figure 13:**
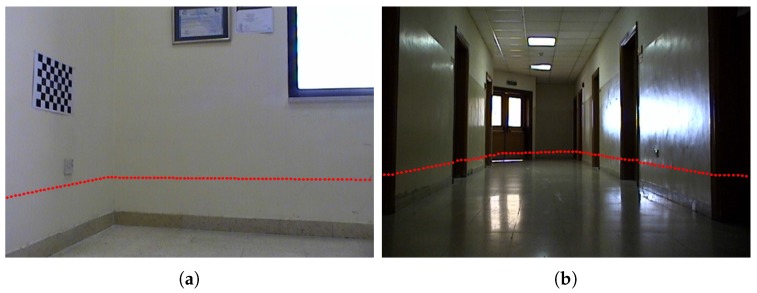
Configuration 2: The projection of the range data to two test images using the estimated Z matrix (points are shown in red color): **(a)** first test image; **(b)** second test image.

**Figure 14:**
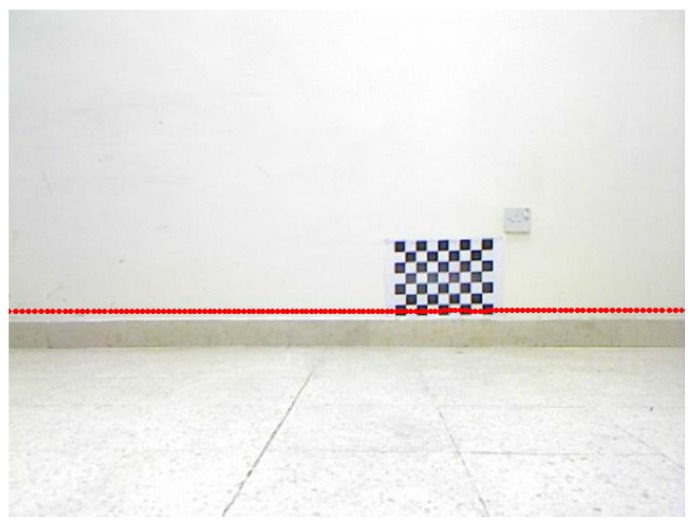
Configuration 4: The projection of the range data to a test image using the estimated Z matrix (points are shown in red color).

Figure 11 and Figure 12 show reprojection error based on the estimated calibration results for configurations 2 and 4, respectively. Blue lines indicate error difference between projected world points and their corresponding original image points. The *rrmse* error values are (in pixels) 3.54517 and 3.10974 for configurations 2 and 4, respectively. Figure 13 and Figure 14 show forward projection of lidar point measurements to selected test images for configurations 2 and 4, which demonstrate high accuracy in the estimated calibration results.

**Figure 15:**
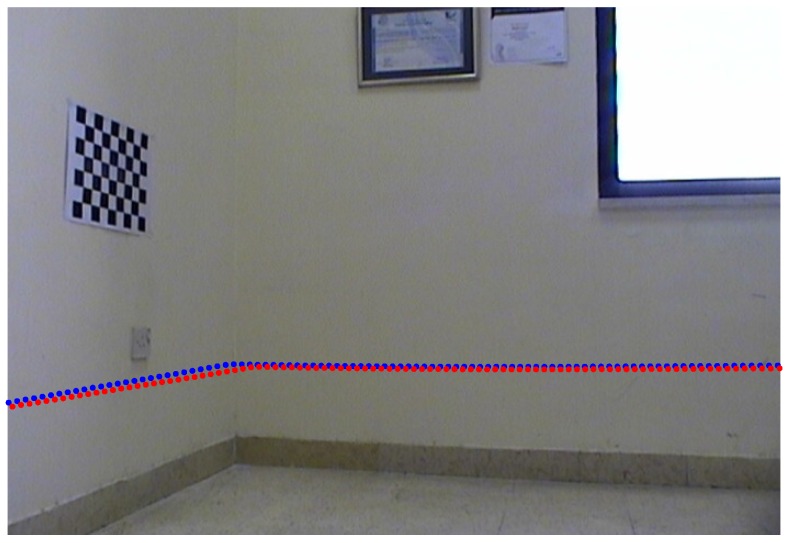
Extra step verification. The blue colored points (upper points) are projection of lidar points onto a test image using the estimated calibration result from configuration 2. The red colored points (lower points) are projection of the same lidar points but using the product of the extrinsic calibration result from configuration 1 and the estimated stereo transformation between the left camera to the PTZ camera.

Additionally, extra verification steps were conducted in configurations 1 and 2 to further demonstrate the accuracy of the estimated calibration results. Specifically, we first performed standard stereo camera calibration using [30] between the left camera of the stereo pair and the PTZ camera sensors, which are mounted on PeopleBot platform and employed in these two configurations. Then, we used the estimated extrinsic calibration parameters for each configuration to project selected lidar point measurements onto their corresponding image planes (these were shown in Figure 9a and Figure 13a). Next, we used the estimated stereo calibration parameters to project the projected lidar points from the left camera (configuration 1) to the PTZ camera (configuration 2). Finally, we computed the average error to the originally projected lidar points on the PTZ camera in configuration 2. The computed average error using the L2 norm is 4.6628 in pixels. Figure 15 demonstrates the results from this experiments, which verify the high accuracy of the proposed calibration approach and results.

### 5.3. Comparison with Zhang and Pless Method

In configuration 2, since the calibration object is in the field of view of the camera and lidar sensors, a comparison between the proposed approach and Zhang and Pless [6] was conducted. In [6], the lidar to camera transformation is determined by fitting the lidar points, which project on the planar calibration object, to the location of the calibration object’s plane as determined by the extrinsic camera calibration parameters. Zhang and Pless estimate one HTM, broken into a rotation matrix (Φ) and translation (Δ) that correspond to the extrinsic transformation from the camera to the lidar sensor. This HTM is analogous to the $Z^{-1}$ matrix in our proposed calibration approach.

Because of differences in collecting the calibration dataset between the two approaches: in our calibration setup:

- the robotic platform cannot be tilted due to platform weight.
- the calibration object is stationary.

Hence, our recored calibration datasets cannot be tested using the the Zhang and Pless approach. Therefore, to address this issue, a new dataset was recorded, where:

- the sensory platform is now stationary,
- the calibration pattern is posed at different views with respect to the sensory platform,
- the recored dataset was collected from 12 poses.

**Figure 16:**
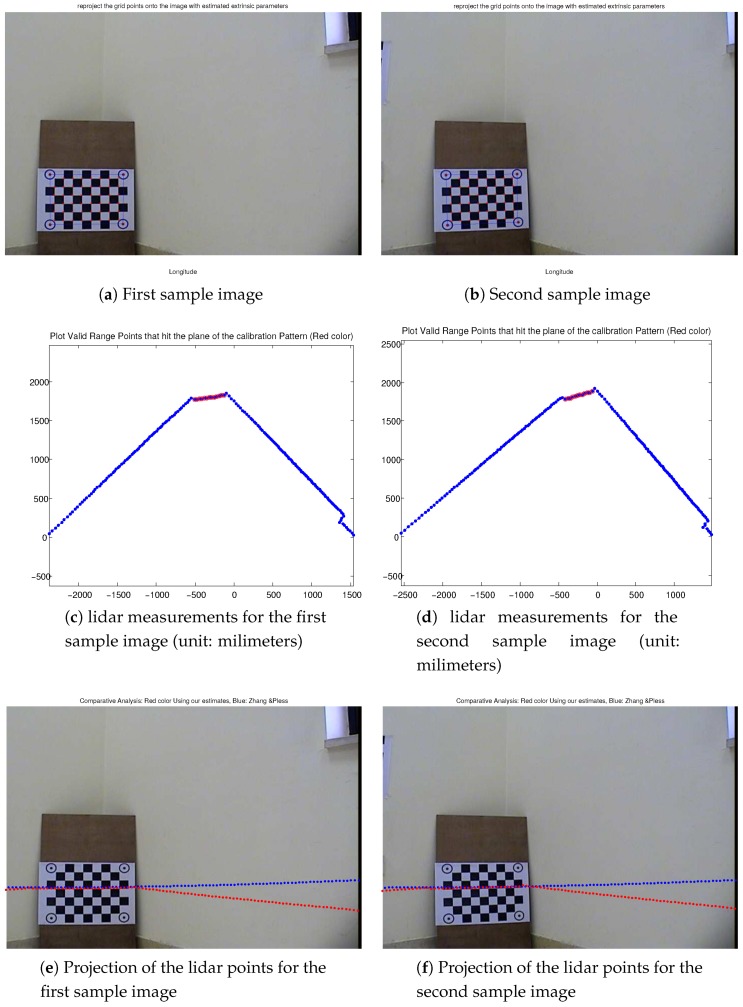
Two sample images and their corresponding lidar measurements from the newly recorded dataset. (**a**,**b**) show the reprojection of the grid points onto the image planes with estimated extrinsic world-camera calibration parameters; (**c**,**d**) show lidar points that were detected in the same plane of the calibration pattern in red color circles, where other lidar points are shown in blue dots (angular resolution is $1^{\circ }$); (**e**,**f**) show forward projection of the lidar points to the image planes using (1) the Zhang and Pless estimated parameters (points shown in blue color) and (2) using the estimates from our proposed approach (points shown in red color).

The newly recorded calibration dataset allowed using Zhang and Pless source code [47]. Figure 16a–d show two sample images and their corresponding lidar measurements from the newly recorded dataset when being supplied to Zhang and Pless method.

The results of comparison between the estimated lidar to camera transformation from the Zhang and Pless approach and the proposed approach are shown in Figure 16e–f, where the comparison is made using the same camera intrinsic parameters and distortion coefficients. Figure 16c,d show the actual lidar points (in red color circles) that went into the estimation of the calibration parameters (those are the actual lidar points on the calibration pattern). Figure 16e,f show forward projection of lidar points to the image planes using (1) the Zhang and Pless estimated parameters (points shown in blue color) and (2) using the estimates from our proposed approach (points shown in red color) for the two sample images in Figure 16a,b.

Figure 16e,f show that the translational and rotational components of both approaches match the shape of the scene only in the vicinity of the calibration pattern or the calibration plane. Overall, our proposed approach performs better in estimating the calibration parameters. Specifically, the rotational components, as computed from our proposed approach, is more accurate especially in the tilting direction when looking at the right wall plane in the images. This can be verified by inspecting the projected lidar points using the estimated parameters from our proposed approach as they tend to be more parallel to the ground plane. The estimated extrinsic transformation computed by our proposed approach is shown in Equation (16), while the transformation computed by the Zhang and Pless approach $\left[\Phi^{-1}\;\;\left(-\Phi^{-1}\Delta \right)\right]$ is shown in Equation (17). The L2 norm of the difference between the translational components of the two transformations is equal to 314.43 mm , whereas the L2 norm of the difference between the Euler angles vector representation of the rotational components is equal to $0.1215^{\circ }$:

(16) $$Z=\left[\begin{array}{cccc} 0.999890 & -0.013132 & -0.006903 & -51.299995\\ -0.005976 & 0.069357 & -0.997574 & 234.444555\\ 0.013579 & 0.997505 & 0.069271 & 48.846367\\ 0.000000 & 0.000000 & 0.000000 & 1.000000 \end{array}\right],$$

(17) $$\left[\begin{array}{cc} \phi^{-1} & -\phi^{-1}\Delta \\ \vec{0} & 1 \end{array}\right]=\left[\begin{array}{cccc} 0.99955 & 0.022982 & -0.019517 & -11.342\\ 0.023362 & -0.18112 & -0.98318 & -76.293\\ -0.019061 & 0.98319 & 0.18157 & 22.173\\ 0.000000 & 0.000000 & 0.000000 & 1.000000 \end{array}\right].$$

### 5.4. Uniqueness of the Proposed Approach

The presented approach in this paper share many similarities that come from both Zhang and Pless [6] and Bok et al. [29]. In this subsection, the added values of our approach are highlighted.

While our proposed approach and that of Zhang and Pless similarly estimate the desired extrinsic calibration parameters, our approach does not require that the camera–lidar sensors share a common field of view of the calibration object. Both approaches estimate the desired extrinsic calibration parameters by minimizing the distance between the plane of the calibration pattern and the location of the pattern as estimated by the camera. However, our approach casts the problem using the RWHE calibration formulation and considers different assumptions about the scene and the calibration procedure. Compared with the Zhang and Pless approach, our calibration paradigm is considered a harder problem because the field of views of the sensors need not overlap, and the moving robotic platform may not be tilted during calibration due to many reasons such as a heavy mobile robot base. The sensory platform weight leads to the problem in that the recorded calibration dataset lacks any excitation in the roll and pitch angles and thus having some of the extrinsic calibration parameters be unobservable. This was successfully mitigated by using manually measured $d_{pattern}$ distance. Additionally, having the robot platform moving against a stationary calibration target allows for automatic or semi-automatic robot platform self-calibration (considering the manually measured distances $d_{pattern}$ and $d_{RF}$), which is not feasible with the Zhang and Pless approach.

Comparing our approach with Bok et al. [29], both approaches deal with no-overlap extrinsic calibration of a lidar–camera systems and also adopt some reasonable assumptions about the calibration environment. However, there are few differences between the two approaches. First, Bok et al. defined either a plane perpendicular to one of the axes of the world coordinate system (as defined by the calibration pattern), or a line intersecting two planes that is parallel to one of the axes of the world coordinate system to perform the calibration. In our approach, we defined a corner on the floor and required that the lidar scans are to be parallel to the ground plane. Second, the approach by Bok et al. requires manual selection of lidar data that overlapped the defined geometric structure. In our proposed approach, the manual selection of lidar data is not required; instead, $d_{pattern}$ and $d_{RF}$ distances are assumed to be manually measured. Lastly, the nonlinear optimization cost function used in the Bok et al. approach is structure dependent, where in our approach the cost function is structure independent and only associated with the employed RWHE calibration approach.

Finally, our proposed approach is flexible and scalable to consider other configurations or setups. For example, one could add many other lidars and cameras and be very flexible with the presented approach to estimate the calibration parameters. The only requirement is to compute the transformation matrices (As and Bs) for the RWHE method to estimate the desired calibration parameters X and Z. In addition, one may need to consider fixing the translational components of the estimated matrices similar to what was done when we fixed the *y* translational component of the ${}^{W}{\hat{X}}_{F}$ matrix in Equation (10) to remove the translational ambiguity.

## 6. Conclusions

This paper presented a novel approach for the extrinsic calibration of a camera–lidar system without overlap based on a RWHE calibration problem. The system was mapped to the RWHE calibration problem and the transformation matrix B was computed, by considering reasonable assumptions about the calibration environment. The calibration results of various experiments and configurations were analyzed. The accuracy of the results was examined and verified. Our approach was compared to a state-of-the-art method in extrinsic 2D lidar to camera calibration. Results indicate that the proposed approach provides better accuracy with an L2 norm translational and rotational deviations of 314 mm and $0.12^{\circ }$, respectively.

The presented work is unique, flexible, and scalable. Our approach is considered one of the few studies addressing the topic of target-based extrinsic calibration with no overlap. We believe that it can easily be a part of an automatic robotic platform self-calibration assuming the required distances (i.e., $d_{RF}$ and $d_{pattern}$) are known. Additionally, we could add other lidars and cameras to the proposed system and still be capable of using the proposed calibration approach.

**Figure 17:**
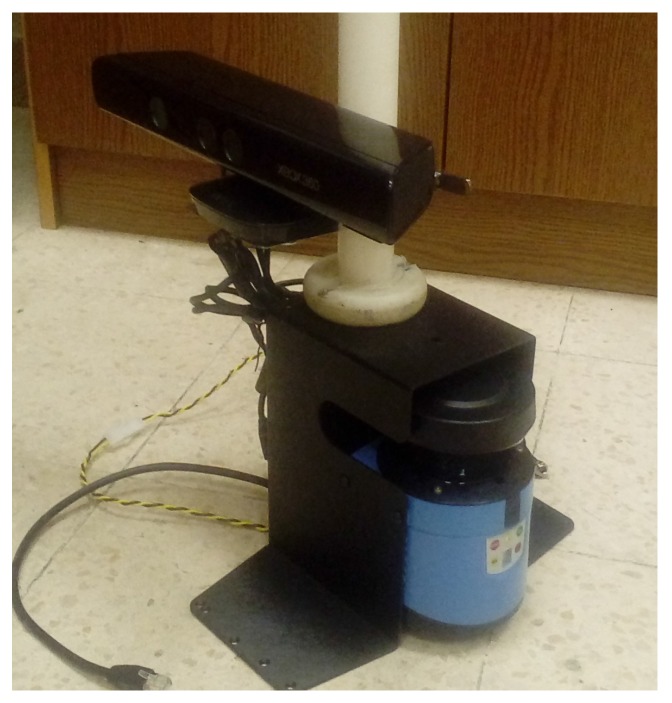
A configuration where the views of the sensors are in different planes.

Future work could consider applying the presented ideas to other robotic platforms with possibly different placements of the heterogeneous sensors after deciding the possible importance of such configurations in the robotic and computer vision communities. For example, Figure 17 illustrates a system of a camera and 2D lidar sensors with a non-overlapping field of view, and the views of the sensors are in completely different planes. Considering the possible sources of uncertainties in the calibration procedure, and thus in the estimated parameters, is another point of future work. Furthermore, future work may possibly include researching the calibration problem when the lidar sensor on the robotic platform is being tilted such that the lidar scans are not being parallel to the ground floor anymore.

## Figures and Tables

**Table 1:** The calibration distances $d_{RF}$ and $d_{pattern}$ for the four configurations in Figure 2.

Measured Distance (mm)	Configuration 1	Configuration 2	Configuration 3	Configuration 4
$d_{pattern}$	830	830	830	160
$d_{RF}$	340	340	140	140
